# Effect of Supportive Supervision on Routine Immunization Service Delivery-A Randomized Post-Test Study in Odisha

**DOI:** 10.5539/gjhs.v6n6p61

**Published:** 2014-06-30

**Authors:** Meena Som, Bhuputra Panda, Sanghamitra Pati, Srinivas Nallala, Anita Anasuya, Abhimanyu Singh Chauhan, Ashish Kumar Sen, Sanjay Zodpey

**Affiliations:** 1 UNICEF State Office for Odisha, India; 2 PHFI-Indian Institute of Public Health- Bhubaneswar, India; 3 DFID-Technical Management and Support Team, Odisha, India; 4 UNICEF State Office for Odisha, India; 5 Public Health Foundation of India, New delhi, India

**Keywords:** Immunization practices, Supportive supervision, Quality of services, Session site

## Abstract

**Introduction:**

Routine immunization is a key child survival intervention. Issues related to quality of service delivery pose operational challenges in delivering effective immunization services. Accumulated evidences suggest that “supportive supervision” improves the quality of health care services. During 2009-10, Govt. of Odisha (GoO) and UNICEF jointly piloted this strategy in four districts to improve routine immunization. The present study aims to assess the effect of supportive supervision strategy on improvement of knowledge and practices on routine immunization among service providers.

**Materials and Methods:**

We adopted a ‘post-test only’ study design to compare the knowledge and practices of frontline health workers and their supervisors in four intervention districts with that of two control districts. Altogether we interviewed 170 supervisors and supervisees (health workers), each, using semi-structured interview schedules. We also directly observed 25 ice lined refrigerator (ILR) points in both groups of districts. The findings were compared with the baseline information, available only for the intervention districts.

**Results:**

The health workers in the intervention districts displayed a higher knowledge score in selected items than in the control group. No significant difference in knowledge was observed between control and intervention supervisors. The management practices at ILR points on key routine immunization components were found to have improved significantly in intervention districts.

**Conclusion and Recommendations:**

The observed improvements in the ILR management practices indicate positive influence of supportive supervision. Higher level of domain knowledge among intervention health workers on specific items related to routine immunization could be due to successful transfer of knowledge from supervisors. A ‘pre-post’ study design should be undertaken to gain insights into the effectiveness of supportive supervision in improving routine immunization services.

## 1. Introduction

The role of continuous supervision in improving quality of health services and its effective management has gathered global momentum. Supportive supervision (SS) as a strategy in the delivery of public health services promotes quality at all levels of the health system through development of professional competence among the health workforce (Rome AK et al., 2005). SS strengthens relationships within the system, focuses on the identification and resolution of problems on the site, optimizes resource allocation, and promotes team work and two-way communication (Marquez & Kean, 2002). The Ministry of Health & Family Welfare (MoH & FW) guidelines, 2005 describe SS as a “process which promotes quality outcomes by strengthening communication, identifying and solving problem, facilitating team work, and providing leadership and support to empower health providers to monitor and improve their own performance” (GOI). In the context of this study, SS is defined as a “Range of measures to ensure that personnel carry out their activities effectively through direct, personal contact on a regular basis”, which in turn, would guide, support and assist designated staff to become more competent at work.

Immunization is one of the safest and most effective interventions in protecting children from vaccine preventable diseases (VPD). GoO has shown its sustained commitment to implementing universal immunization programme (UIP) as a key child survival strategy (INCLEN, 2004). Emerging evidences in Indian context infer that supportive supervision improves immunization coverage and also serves as an efficient tool to strengthen the local health system (Babu et al., 2010). Another review reported that community health workers (CHW) make diverse contributions toward strengthening immunization program implementation by incorporating evidence- based strategies (Patel & Nowalk, 2010).

The national UIP review (2004) and Vaccine Management Assessment Tool (VMAT) assessment (2007) studies identified ‘inadequate supportive supervision and poor capacity building measures’ by the government as foremost gaps underlying poor coverage and quality of immunization (UNICEF, 2007). In order to reduce the urban-rural divide of antigen-wise coverage and to further improve upon the coverage and quality of services, the GoO, in collaboration with UNICEF, adopted SS as an important strategy and imparted training on SS to the district and sub-district level supervisory cadre in four selected districts during Aug. 2009–Feb. 2010.

GoO in partnership with the UNICEF piloted “*Routine Immunization Assessment cum Training Workshops on Supportive Supervision”* for the supervisors of routine immunization (RI) programme in four high priority districts -Bolangir, Koraput, Malkangiri and Nabarangpur. About eight to ten participants from each block of these districts attended this training during Nov 2009–Feb 2010 in batches of 24 participants. Medical officers (MO), AYUSH medical officers (AYUSH MO), block extension educators (BEE), block programme organizers (BPO), lady health visitors (LHV), integrated child development scheme (ICDS) supervisors and supervisors at block level of the women and child development (WCD) department. The content of such training workshops consisted of components, such as, development of supportive supervision guidelines, monitoring and evaluation of performance, effective communication, allocation of resources and evidence based planning. Full-scale implementation of this pilot initiative was reported since May 2010. The subsequent 12 months involved extensive monitoring and on-the-job training of immunization managers and supervisors to improve supervision practices and to help providers solve immunization-related problems. Immunization managers from four intervention districts were encouraged to apply supportive supervision guidelines in practice. Guidelines and tools for supervision included detailed instructions for conducting supervision, namely, rules of conducting supervision meeting, checklist for supervisory visit, work planning action sheets, do’s and don’ts of supervision, self-assessment of supervisors’ competencies, tips on delegation, feedback mechanism and conflict resolution. It was mandatory for every immunization manager from the intervention group to visit sessions at least once in a week during the fixed outreach session (UNICEF, 2009).

This study aimed to assess the effect of earlier interventions in terms of improvement in the knowledge and practice level of supervisees as well as of the supervisors, with special focus on micro-plan preparation, cold-chain maintenance, availability of supplies and vaccine logistics, injection safety and waste disposal. The specific objectives of the study were: a) to assess and compare the level of knowledge, opinion and perception of supervisor-supervisee pairs; b) to observe key quality parameters at ILR points of only intervention districts and compare with the base-line report; and c) to recommend to the state government and to UNICEF about usefulness of supportive supervision strategy in improvement of quality of immunization services.

## 2. Materials and Methods

### 2.1 Study Design and Settings

We adopted a quasi-experimental ‘post test-only’ study design. It focused mainly on the assessment of technical knowledge, role clarity and practices at the immunization site of supervisors and supervisees. In Odisha context, the supervisees were multi-purpose health workers female, also known as auxiliary nurse and midwives (ANM) and Anganwadi workers (AWW). Four pre-existing intervention districts (ID) and two comparable non-intervention districts, in other words termed as control districts (CD) were taken for data collection. The decision to include only two CDs was taken in view of resource constraints. Selection of CD was done on the basis of comparable base-line UIP indicators as on 31^st^ Dec, 2009, through desk review. On the basis of geographic and socio-political similarity, Kandhamal, and Kalahandi were chosen as control districts. All supervisors who had undergone SS training and posted in the intervention districts during data collection were included in the sample frame, while supervisors who had undergone the SS training but were transferred or retired or were absent during data collection were excluded. On an average, a supervisor from health & family welfare department (DH&FW) oversees the activities of about 4-6 ANMs and a supervisor from WCD looks after about 20-25 Anganwadi workers (AWW).

### 2.2 Sampling

For objective 1, we included about 30 percent of total trained, eligible and available supervisors and equal number of supervisees in the sample. All supervisors were first enlisted with the support of latest database from the concerned districts. Multi-stage stratified random sampling technique was used to select the sample supervisors. In the next step, from among the list of supervisees that each supervisor had overseen the work at least six times in last one year. We identified the supervisees randomly and tagged into supervisor-supervisee pairs. This supervisor-supervisee pair was taken as one unit of sample. Total 109 samples from intervention districts (33% of supervisors) and 61 from control districts (35% of supervisors) were taken in pairs (170 X 2 = 340). For objective ll, direct observation of 25 ILR points was conducted which constituted about 30% of all operational ILR points across these four districts. Selection of ILR points was done at random.

### 2.3 Data Collection Tools

For objective l, a semi-structured interview schedule was prepared separately, each, for supervisors and supervisees, though both schedules had many questions in common. It aimed to test the knowledge/opinion of basic health workers and supervisors. Researchers from Indian institute of public health–Bhubaneswar (IIPHB) and field investigators visited two ILR points for field-testing the tools. It was subsequently translated into Odiya. Interview of supervisors and supervisees were conducted at sub centres and primary health centres, block headquarters or community health centres on mutual convenience. For objective ll, a direct observation checklist, extracted from the government of India format, was prepared to assess the quality parameters at ILR points-the hub of logistics and supply chain management for RI. The checklist consisted of questions related to availability of vaccines and logistics, compliance with micro-plan, scheduling of sessions and practices of supervisors with regard to temperature maintenance, cold chain management, indenting and supply of vaccines and logistics, etc.

### 2.4 Data Analysis

Quantitative data was entered into M.S Excel and then exported to SPSS 16.0 for analysis. Questions on knowledge scales were analyzed through derivation of means; statistical techniques, such as, chi-square and t-tests were used to assess the association levels. While analyzing data, P value of less than < 0.05 was considered statistically significant and <0.001 as highly significant. Sub-group analysis of macro management components was carried out by categorizing the intervention districts in to ‘Koraput’ and ‘non-koraput’ regions and compared with the CD results. This categorization of ID into two further sub-groups (Koraput and non-koraput) was done for selected indicators against which the base-line information was also available.

### 2.5 Ethical Issues

We obtained ethical approval of the study from an independent ethical committee. Confidentiality of data and anonymity of respondents was maintained throughout the study. Informed consent of the all ILR in-charge officers was taken for direct observation. Written consent of all respondents was obtained for conducting interview. They were briefed about the study objectives. The respondents were free not to respond to any question or leave the interview at any stage. All field investigators were hired, trained and deployed for data collection after developing common understanding on the tool. Researchers also visited the ILR points and interview sites to cross check authenticity of data being collected by field investigators.

## 3. Results

### 3.1 Supervisors

Comparison of knowledge level among supervisors in intervention and control districts was done through quantitative analysis. Results indicated that the level of knowledge and understanding among the supervisors of the intervention districts was almost the same as that of control districts: the mean score of the former was 5.76 as compared to 5.75 in the later and this difference was statistically not significant (*p =* 0.981) (Table 1). Further analyses revealed that the supervisors of intervention districts as compared to their CD counterparts had better knowledge on components, such as, the correct manner of placing ice packs inside DF, storage techniques of RI vaccines and calculation of cumulative drop-out rates. With respect to maintenance of appropriate cabinet temperature at ILR points, both the groups performed equally well. However, the control group members performed better on questions, such as, distance of ILR and DF from the wall, placing of diluents, and calculation dropout and leftout rates.

**Table 1:** Knowledge of Supervisors in intervention and control districts

Item	Correct Response	ID - CRR Percent (N=109)	CD-CRR Percent (N=61)	Chi-square value
Distance of ILRs and DFs from wall	≥ 10 cm	77	83.6	1.12
Temperature log book maintenance rate/day	Minimum two times/day	89	96.72	3.53
Appropriate Cabinet temperature for ILRs	+2 to +8 C	83.0	83.6	3.41
How many hours before distribution, diluents to be placed in ILR point	24 hrs	56.8	70.49	3.64
Correct manner of placing Ice packs inside DF	Crisscross	80.7	62.29	8.85*
Some RI vaccines should be stored inside deep freezers for two hours before taking to ILR	No	97.24	91.8	3.191
Question related to drop-out & left-out	40, 20	61.46	77.04	5.9*
Ability to calculate cumulative drop-out	Able to calculate	29.35	9.83	11.4**

N.B-ILR: Ice Lined Refrigerator; DF: Deep Freezer; CRR: Correct Response Rate; RI: Routine Immunization.

* *p*=<0.05,

** *p*=<0.001.

### 3.2 Supervisees

We found in a scale of 0-6, there was an overall improvement in the level of knowledge and understanding among the supervisees of the ID [mean = 3.39 (2.89-3.92] as compared to CD [mean = 2.52 (2.29-2.86]. This difference was statistically significant (0.037). On further comparison of knowledge level with respect to specific attributes, we found the supervisees of intervention districts had better knowledge on correct maintenance of temperature log book, cabinet temperature, placing of diluents and manner of placing ice packs inside DF. The level of significance on these questions ranged from ‘significant’ to ‘highly significant’. However, on questions, such as, ‘distance of ILRs and DFs from wall’ the performance of both ID and CD group of respondents was comparable (Table 2). Results on knowledge and practices of supervisors and supervisees were compared at macro-level by grouping specific questions into broad management themes. We found there was a significant performance difference between intervention and control districts.

**Table 2:** Knowledge of supervisees in intervention and control districts

Item	Correct Response	ID-CRR Percent (N=109)	CD-CRR Percent (N=61)	Chi-square value
Distance of ILRs and DFs from wall	≥ 10 cm	40.36	40.98	0.021
Temperature log book maintenance rate/day	Minimum two times/day	75.22	62.29	3.91*
Appropriate Cabinet temperature for ILRs	+2 to +8 C	46.78	19.67	16.3**
How many hours before distribution, diluents to be placed in ILR point	24 hrs	41.28	18.03	12.71**
Correct manner of placing Ice packs inside DF	Crisscross	41.28	22.9	7.44
Some RI vaccines should be stored inside deep freezers for two hours before taking to ILR	No	94.49	88.52	1.6

N.B - ILR: Ice Lined Refrigerator; DF: Deep Freezer; CRR: Correct Response Rate; RI: Routine Immunization.

* *p*=<0.05,

** *p*=<0.001

### 3.3 ILR Points

The main purpose of direct observation of ILR points was to validate changes taken place in last two years, after the intervention, in the practice of supervisors with respect to basic management functions at the ILR point level. The base-line data had been collected before the intervention and was available with UNICEF for the ILR points visited during the pilot intervention programme. The checklist used in this study was similar to the base-line checklist used during pilot study. As a result, all questions of pre- and post- were similar, though not identical. Therefore, only the common questions were analyzed and grouped into themes for comparison.

Data on pre-intervention were extracted from the previous published report and compared with the data collected from the same sites during this study, after the intervention (UNICEF, 2010). We analyzed data by breaking into four broad themes of program management, reporting and documentation, cold chain and logistics, and waste management. Sub components under each of these themes are representative of specific attributes pertaining to the question, as reflected in percentage of desirable responses (Table – 3). A statistically significant difference in the performance of the intervention districts before and after the intervention was found in all the broad thematic areas, especially with regard to two sub-components i.e. ‘Reporting & documentation’ and ‘Cold chain & logistics’. The ILR points of intervention districts have significant improvements on reporting and maintenance of logistics after the intervention. For instance, day wise plan preparation for supervisors, display of ANM roasters, period of checking of temperature log-book, ILR temperature maintenance, DF temperature maintenance, correct alignment of icepacks, proper stock register maintenance, reporting of adverse events following immunization (AEFI) and availability and use of disposal pits were all near to 100% during this study as against less than 50% during the base-line.

**Table 3:** Pre and Post Comparison of ILR performance in Intervention districts on key SS components

Specific Attributes	Pre (Avg.) %	Post (Avg.) %
Day-wise Plan for Supervision	38	52
ANM Roster displayed	59	62
Coverage Monitoring Chart displayed	51	72
Periodic checks of Temperature Log Books	53	100
ILR temperature +2 C to +8 C	89	100
No items other than vaccines in ILR	88	100
OPV within usable stage of VVM	97	96
DF Temperature -15C to -18C	81	96
Ice packs correctly arranged in DF	59	80
Vaccine issue register matches Stock Register	49	96
Sessions conducted as planned >80%	94	92
AEFI reported or Zero Report	33	100
VPD reported or Zero Report	31	100
Disposal pit available	51	100
Disposal pit used for disposal of sharps	35	72

N.B – ILR: Ice Lined Refrigerator; OPV: Oral Polio Vaccine; VVM: Vaccine Vial Monitor; AEFI: Adverse Events Following Immunization; VPD: Vaccine Preventable Diseases; ANM: Auxiliary Nurse and Midwife.

## 4. Discussion

For decades it was assumed that poor performance in service delivery was simply due to lack of knowledge and skills. As a result, most interventions concentrated on training; but this had mixed and sometimes disappointing long-term results (Djibuti Mamuka et al., 2009). Reviews of intervention studies in low and middle income countries suggest that formal and informal trainings, regular supervision with feedback is generally more effective than simple dissemination of written guidelines (Esmail, Cohen & Djibuti, 2007). Supervisors often lack the technical, managerial, or supervisory skills needed to effectively evaluate health facilities across sectors for which they are responsible (Drach-Zahavy, 2004). In addition to assessing performance, supervisors are also expected to monitor services, evaluate management, and ensure that the health facility supply chains are working properly (Kroeger & Hernandez, 2003). Consequently, they are unable to provide adequate technical guidance and feedback to improve service delivery. Our study findings are a reflection of the possible poor quality of supervision and the limited time that supervisors provide in delivering RI services.

The knowledge level of the supervisors in the intervention districts is comparable to those of control districts; but there is a significant difference in the knowledge level of the supervisees of intervention and control districts. The supervisees of intervention districts had remarkably higher level of knowledge than their counterparts in control districts. Therefore, it appears that the transfer of knowledge from supervisors to supervisees in intervention districts was higher than that of the control districts; in other words, the supervisors of intervention districts had better transferred the knowledge to their supervisees. Among the supervisors of intervention districts, there was almost no difference in their knowledge level before and after the intervention. One possible explanation to this observation is that the questions asked to supervisors were fundamental of immunization programme and therefore all supervisors irrespective of their training status had similar residual knowledge on the programme; there could hardly have any scope for further improvement since majority of the components already had about 90% performance level. The possible transfer of trained supervisors, over a period of last three years, to control districts could also have resulted in this insignificant knowledge difference. The other most important factor behind comparable performance indicators among supervisors of both categories of districts could be transfer of trained officials to outside intervention districts (but not to control districts), thereby creating a spill-over bias of the study findings. This needs further investigation and was outside the mandate of this particular study. There could be a possibility that untrained personnel have been transferred from intervention to control districts.

Studies across the globe reveal that supportive management and supervision improves nursing performance, increases the health services efficiency (in terms of best use of resources) and equity (in terms of health care provision according to people’s needs), achieve a substantial reduction of the burden of disease at reasonable cost (Djibuti, 2003; Jacobson et al., 1987; Dohlie et al., 2002). Similarly, a controlled field trial, conducted to examine whether systematic supervision using an objective set of indicators could improve health worker performance, concluded that systematic supervision using clearly defined and quantifiable indicators can improve service delivery considerably, at a modest cost (Bradley et al., 2002). Another study documented that intense supervision led to high provider performance in systematic influenza and pneumococcal vaccination in a busy public emergency department, despite initial resistance and extreme variation in individual performance (WHO, 2006).

Directly observed ILR points only in intervention districts (as base-line was available there) revealed significant improvements in their performances. Such kinds of capacity building programmes, thus, ought to be conducted in a real field-setting and with periodic follow-ups for ensuring long-term results.

## 5. Limitations

The individual level data are subjective and socially desirable responses could have biased the study results. The questions used might not have been sensitive enough to capture changes in knowledge parameters at micro-level. The sample size to observe a clinically significant difference couldn’t be calculated due to non-availability of base-line information; this might have affected the statistical power of the study.

## 6. Conclusion

Functional knowledge level of supervisors engaged in supervising immunization services in Odisha was uniformly good across all six districts. Those supervisors who had undergone the rigorously planned capacity building and demonstration exercise in the field were better able to transfer knowledge and skills to the supervisees. Improvements in the ILR points were associated with the intervention. Management of human resources, especially with regard to their training needs, transfers and performance assessment may be done in a guided manner. Those trained on SS may be placed to supervise the most hard-to-reach areas with incentives to sustain their motivation level.

## References

[ref1] Babu G, Singh V, Nandy S, Jana S (2010). Supportive Supervision And Immunization Coverage: Evidence From India. Int J Epidemiol.

[ref2] Bradley J, Igras S, Shire A, Diallo M, Matwale E, Fofana F (2002). COPE®for Child Health in Kenya and Guinea: An Analysis of Service Quality.

[ref3] Centers for Disease Control and Prevention (CDC) (2011). Ten great public health achievements--worldwide 2001-2010 MMWR. Morbidity and mortality weekly report.

[ref4] Djibuti M, Chikovani I, Zakhashvili K, Gotsadze G (2003). Knowledge attitudes and behaviors toward VPD surveillance among health care providers and community members in Georgia. Focus group discussion report.

[ref5] Drach-Zahavy A (2004). Primary nurses’ performance: role of supportive management. Journal of Advanced Nursing.

[ref6] Esmail L, Cohen J. C, Djibuti M (2007). Optimizing Human Resource Management: Lessons from the Georgian National Immunization Program. Hum Resour Health.

[ref7] Georgia Primary Health Care Development Project, Ministry of Labor Health and Social Affairs (2007). Georgia Health Utilization and Expenditure Survey, Final Report.

[ref8] Government of India. (n.d.) Child Health Division, Department of Family Welfare, Ministry of Health & Family Welfare, Introduction of Hepatitis B.

[ref9] Haberland N, Measham D (2002). Responding to Cairo: case studies of changing practice in reproductive health and family planning. Population Council.

[ref10] INCLEN. (n.d.) Evaluation of Universal Immunization Program in India (2004-2005): An IndiaCLEN Program Evaluation Network Study 2004.

[ref11] Jacobson M. L, Labbok M. H, Murage A. N, Parker R. L (1987). Individual and group supervision of community health workers in Kenya: a comparison. Journal of Health Administration Education.

[ref12] Kroeger A, Hernandez J. M (2003). Health services analysis as a tool for evidence-based policy decisions: the case of the Ministry of Health and Social Security in Mexico. Tropical Medicine & International Health.

[ref13] Mamuka Djibuti, George Gotsadze, Akaki Zoidze, George Mataradze, Laura C Esmail, Jillian Clare Kohler The role of supportive supervision on immunization program outcome-a randomized field trial from Georgia. http://www.ncbi.nlm.nih.gov/pmc/articles/PMC3226230/.

[ref14] Marquez L, Kean L (2002). Making supervision supportive and sustainable: new approaches to old problems. www.maqweb.org/maqdoc/MAQno4final.pdf.

[ref15] Patel A. R, Nowalk M. P (2010). Expanding immunization coverage in rural India: a review of evidence for the role of community health workers. Vaccine.

[ref16] Rowe A. K, de Savigny D, Lanata C. F, Victora C. G (2005). How can we achieve and maintain high-quality performance of health workers in low-resource settings?. The Lancet.

[ref17] UNICEF (2009). Report on Supportive Supervision for Routine Immunization in Odisha.

[ref18] UNICEF (2010). Report on Implementation of Routine Immunization in Odisha.

[ref19] UNICEF. (n.d.) Assessment cum training of vaccine and cold chain management in Odisha. A VMAT Study 2007. http://www.unicef.org/india/VMAT_Orissa.pdf.

[ref20] World Health Organization (2006). WHO recommendations for clinical mentoring to support scale-up of HIV care, antiretroviral therapy and prevention in resource-constrained settings.

